# An optimised eDNA protocol for detecting fish in lentic and lotic freshwaters using a small water volume

**DOI:** 10.1371/journal.pone.0219218

**Published:** 2019-07-17

**Authors:** Teja Petra Muha, Chloe Victoria Robinson, Carlos Garcia de Leaniz, Sofia Consuegra

**Affiliations:** Swansea University, Department of Biosciences, Singleton Park, Swansea, Wales, United Kingdom; University of Hyogo, JAPAN

## Abstract

Environmental DNA is increasingly being used for assessing the presence and relative abundance of fish in freshwater, but existing protocols typically rely on filtering large volumes of water which is not always practical. We compared the effects of water volume, filtration type and eDNA extraction procedures in the detection of fish in three freshwater bodies (pond, lake and river) using a short fragment of the 12s rRNA mtDNA gene. Quantification of eDNA capture efficiency after DNA extraction, as well as amplification efficiency, were evaluated by conventional PCR and quantitative PCR. No significant differences on eDNA capture yield were found among freshwater bodies, but increasing water volume had a positive effect on eDNA capture and amplification efficiency. Although highest eDNA capture rates were obtained using 2 L of filtered water, 100 mL syringe filtration in combination with ethanol- sodium acetate precipitation proved to be more practical and increased quantitative PCR amplification efficiency by 6.4%. Our results indicate that such method may be optimal to detect fish species effectively across both lotic and lentic freshwater environments.

## Introduction

Environmental DNA (eDNA) is increasingly being used in freshwater environments to detect the presence of target invertebrate and vertebrate species, based on the detection of short extracellular DNA fragments released into the environment [1–3]. eDNA detection can be used for management purposes, such as monitoring of species’ presence/absence [1], invasive species detection [4], relative abundance estimates [5] and use of space [6]. In some cases it can offer more efficient estimations of relative abundance than conventional sampling techniques [7] as it can provide higher detection sensitivity [8]. Examples of accurate eDNA presence/ absence detection rates include the American bullfrog *Lithobates catesbeianus* [9], the smooth newt *Lissotriton vulgaris* [10] and the great crested newt *Triturus cristatus* [11].

The analysis of eDNA allows to identify particular target species or even the composition of an entire community, by using either barcoding or metabarcoding approaches. Species specific eDNA assays can be used to estimate the density of species, through the relationship between their abundance and the detection rate of their eDNA [12–14], mainly by quantitative PCR (qPCR) [15, 16]. On the other hand, eDNA metabarcoding takes advantages of the ability of next generation sequencing (NGS) techniques to sequence short fragments of DNA [17] and assign the sequences to its corresponding taxon, potentially allowing to assess relative abundance based on within target species comparison [5].

Several studies have focused on the benefits and limitations of different eDNA techniques [1, 18–21], and a number of comparative approaches have tested the efficiencies of eDNA capture by ethanol precipitation or filtration [20, 22], methods of preservation [23, 24], filter types and extraction kits [25]. It has been established, for example, that the efficiency of eDNA sampling techniques differs between lentic and lotic bodies, based on a study of four different invertebrate species using species specific primers [20]. Yet, a consistent application of the same eDNA protocol across water bodies for species detection [26, 27] or relative abundance purposes [28, 29] is still lacking.

Two of the most widely employed techniques of eDNA capturing are the ethanol- sodium acetate precipitation [30] and the filtration method [31–33]. Ethanol precipitation allows a wider size range of eDNA detection, whereas filtering largely depends on the pore size [34]. Glass fibre filters [26, 31, 35] and cellulose nitrate filters [29, 36, 37] with different pore sizes are the most commonly used filter materials in eDNA studies. Both methods (precipitation and filtering) have shown variable success rates in comparative studies [22, 34], depending on the volume of water, pore size, filter material and extraction methods used, in addition to environmental and physical conditions [20, 22, 38]. For example, ethanol- sodium acetate precipitation is unfeasible for large water volumes whereas the efficiency of the filtration largely depends on the turbidity of the water filtered water, resulting in different eDNA capture success rate. Environmental conditions in lotic bodies, particularly the acidity, can accelerate eDNA decay, affecting its abundance [39]. Thus, eDNA extraction using ethanol- sodium acetate precipitation tends to be done on small (15 mL) water samples [7, 9, 30] and appears suitable when target species are highly abundant (and hence there is a lot of eDNA) in small or closed freshwater systems [34], whereas filtration of larger volumes of water seems to be more efficient in larger systems. The type of eDNA extraction kit also determines overall eDNA capture rate efficiency [20, 40–42] but this can vary depending on the presence of inhibitors [42, 43] and pollutants that can increase the number of extraction steps, and unintentionally provide false positives by increasing exposure to potential contamination [44].

A fully optimised method should have low contamination risk and ideally allow the sampling of different water bodies. Using syringe filters in combination with ethanol precipitation, for example, could reduce the risk of contamination at the start of the eDNA processing pipeline [44]. We carried out a comparison of different methods of eDNA collection and extraction in both lentic and lotic freshwaters, using vertebrate specific primers (avoiding species specific variation in eDNA shedding, detection and amplification and ensuring amplification of fish which were expected in all of them) to assess the importance of three key factors that determine eDNA capture efficiency, namely water volume, filtration method and DNA extraction kit.

## Material and methods

### Study sites

Water samples of various volumes were collected in April 2017 from three freshwater bodies (two lentic and one lotic) in Wales (UK): a small (15 m wide, 1 m deep) pond located at Swansea University, an artificial freshwater lake at Cardiff Bay and the River Tawe. Cardiff Bay is situated at the confluence of the Rivers Taff and Ely, it is approximately 200 ha and was impounded in 1999 [45]. Water from the River Tawe was collected at the headwaters, close to the river source (latitude 51°46’0.276” N, longitude 3°46’35.514” W), and also at the river mouth (latitude 51°42'08.9"N, longitude 3°53'57.2"W). In the pond, water was collected at two different sampling points on opposite sides (longitude 51°36'26.5"N, latitude 3°58'52.5"W). The water samples in Cardiff Bay were collected from three different stations; the barrage (longitude 51°26'48.7"N, latitude 3°09'59.4"W); St David’s Hotel (longitude 51°27'39.1"N, latitude 3°10'01.1"W) and Cardiff International White Waters (longitude 51°26'52.6"N, latitude 3°10'57.1"W).

### eDNA sampling procedure

Three replicates were obtained from each water body, collected approximately 30 cm under the surface. Water samples were kept refrigerated and transported to the laboratory for filtration within four hours of collection. To minimize the risk of cross-contamination, disposable nitrile gloves were used and Nalgene polyethylene bottles were treated with 10% bleach, left for 5 min and thoroughly rinsed with sterile distilled water before sampling at each station. All filtration was conducted on the day of the sampling. Water was thoroughly mixed between sampling stations before filtration in order to have one uniform representation for each specific water body.

### eDNA capture and amplification efficiency experiment

**Fig 1:**
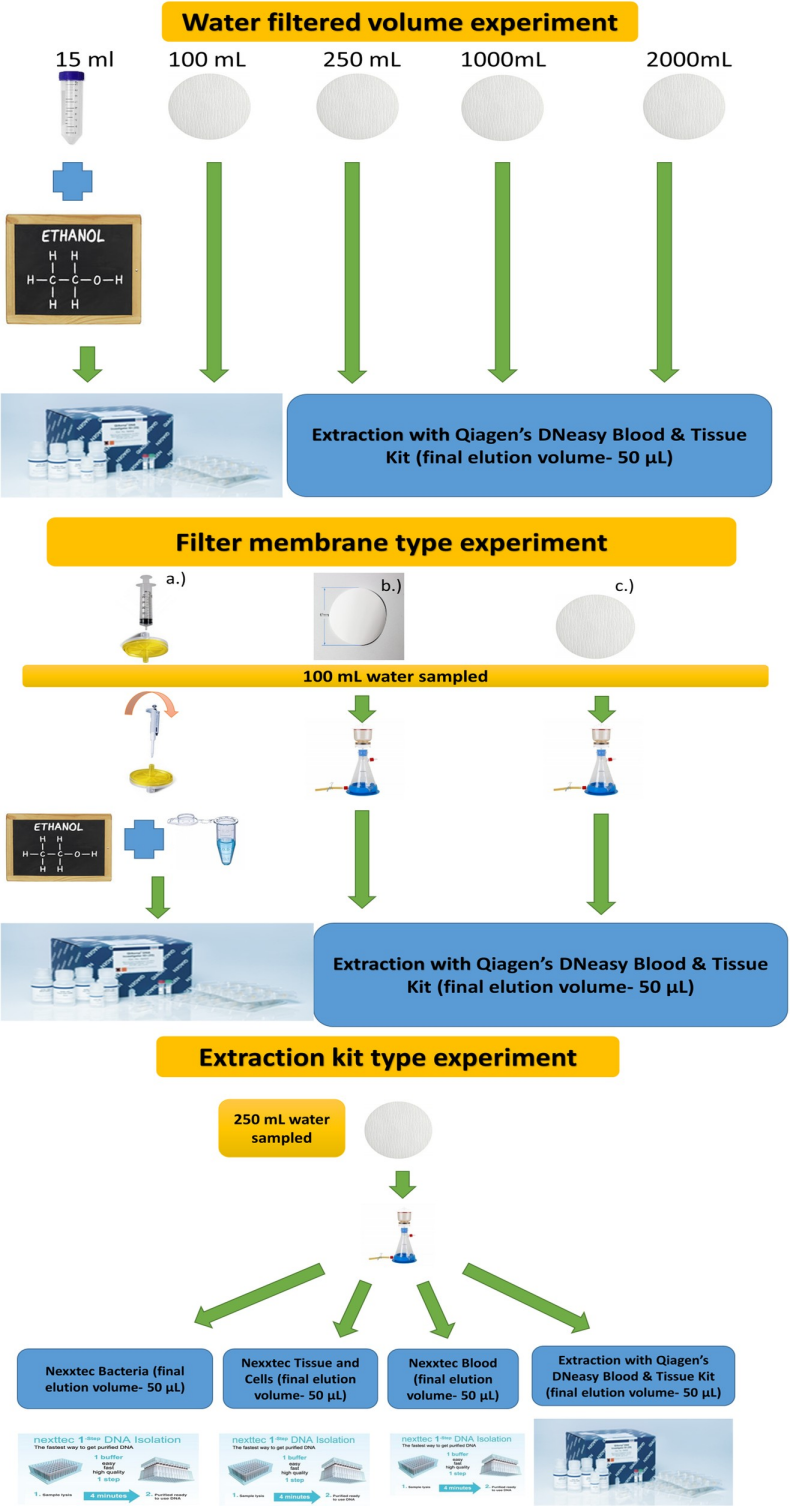
Graphical representation of filtration volume, filtration type and extraction kit experiments.

The study evaluated the effects of different filtered volume, filter type, and extraction kits (Fig 1) individually. The efficiency of the experiments was assessed by eDNA capture yield (ng/μ*L*), PCR amplification (ng/μ*L*) and amplification using qPCR (Cq values). DNA yield as well as the efficiency of PCR amplification were measured using a Qubit 1.0 fluorometer (Thermo Fisher Scientific Inc., UK) applying the high-sensitivity assay for DNA capture yield efficiencies and broad range assay for PCR products (Life Technologies, Carlsbad, CA, USA). Standard recommendations for work with eDNA were applied through all the study [1]. In total, 108 field samples were extracted, plus six and nine filtration and extraction control samples, respectively. The extraction and pre-PCR handling of eDNA water samples was carried out in a fume hood dedicated to eDNA analyses only. Before individual extractions, 10% bleach was used to clean up the fume hood as well as 45 min exposure to UV light.

The water volume experiment included 15 mL of volume without filtration based on ethanol- sodium acetate precipitation with the rest of volumes (100 mL, 250 mL, 1000 mL and 2000 mL) based only on filtering. All filtration was carried out using glass fibre filter and DNA was extracted using Qiagen kits. The filtration type experiment was based on 100 mL of water and DNA was extracted using the Qiagen extraction kit, comparing ethanol- sodium acetate precipitation with cellulose filtration and two other filter types, cellulose nitrate and glass fibre, without precipitation. The extraction kit experiment compared the Nexxtec Bacteria, Tissue and cell kit, the Nexxtec Blood kit and the Qiagen DNeasy Blood & Tissue kits, filtering 250 mL of water with glass fibre filter.

### Water filtration volume comparison

For comparisons of water filtration volumes, three replicates of 15 mL, 100 mL, 250 mL, 1000 mL and 2000 mL water, were collected at each site (Fig 1). For the 15 mL water samples we followed the protocol for ethanol- sodium acetate precipitation described in [30] by adding 1.5 mL sodium acetate (3 M) and 33 mL of absolute ethanol. The mixture was centrifuged at 5000 g for 45 min at 6°C and the supernatant was discarded [46]. The precipitation itself was conducted on the day of water sampling, by centrifuging no more than four hours after collection. The falcon tubes with the DNA pellets were then stored at -20°C without preservatives until the DNA extraction one week later. Negative control nuclease-free water was included.

The larger water volumes (100 mL, 250 mL, 1000 mL and 2000 mL) were filtered through the Advantec GA55 Borosilicate Glass Fibre Filters with 0.6 μm pore size (47 mm) (Fig 1). Each water sample was filtered through a filter funnel attached to a collection bottle and connected to the electronic vacuum pump Welch N. 2522C- 02, with strength of 20 kpa for 15 s up to 75 s per sample. To avoid contamination, the filter funnel and handling tweezers were cleaned with a 10% bleach solution, rinsed with 99% molecular grade ethanol and then with sterile nuclease-free water between samples. For each different volume, a negative control consisting of nuclease-free water was used. For eDNA extraction, the Qiagen DNeasy Blood & Tissue DNA extraction kit (Qiagen GmbH, Hilden, Germany) was used. For the 15 mL method the Qiagen protocol for blood (spin protocol) was used whereas for the filtration methods we used the protocol for dried blood spots. The manufacturer’s protocol was followed in all cases, with the single modification of reducing the final elution volume to 50 μL in all three experimental designs. In total, 45 samples were used for water filtering comparison, 15 per water body, 3 for each of the five volume categories, with all samples amplified in duplicate for PCR and qPCR (Table B in S1 File).

### Filtration type comparison

For the filtration type comparison, we used 100 mL of water and two different DNA capture methods: a pump filtration only method and syringe filtration with additional ethanol- sodium acetate precipitation. For the filtration only method, we used two different filter materials, Whatman Cellulose Nitrate Membrane Circle filters with 0.45 μm pore size (47 mm) and Advantec GA55 Borosilicate Glass Fibre Filters with 0.6 μm pore size (47 mm) (Fig 1). The second capture method was based on a combination of filtration using closed syringe filters Minisart cellulose syringe filters with 0.45 μm pore size (Sartorius, Germany) with additional ethanol- sodium acetate precipitation. For the syringe filtration, the water was pushed through by hand at an approximate flow rate of 50 mL per 30 s. After filtration, a mixture of 1350 μL absolute ethanol and 150 μL of sodium acetate was passed through the filters which were then centrifuged at 5000 g for 45 min at 6°C. For the other two types of filters, filtration was carried out as above. DNA was purified with the Qiagen DNeasy Blood & Tissue DNA extraction kit. For the 100 mL syringe filtration method, the Qiagen DNA purification protocol for blood (spin protocol) was used, whereas for the other two filtration techniques we applied the protocol for dried blood spots, designed for the DNA isolation out of filter paper. In total, 27 samples were extracted for comparison, 9 per water body, 3 per each of the three filtration comparison types, all samples analysed in duplicates at both PCR and qPCR (Table C in S1 File).

### Extraction kit comparison

Two hundred and fifty mL of water were collected and filtered through Advantec GA55 Borosilicate Glass Fibre Filters with 0.6 μm pore size (47 mm) for the extraction kit comparison (Fig 1). The 250 mL water volume was selected for practical reasons, as larger volumes result in reduced estimate error for all replicates [29]. The Qiagen DNeasy Blood & Tissue DNA extraction kit (protocol for dried blood spots) was compared to three additional kits all from Nexttec (Nexttec Biotechnologie GmbH, Germany): the 1-step DNA Isolation Kit for Tissues & Cells, 1- step DNA Isolation Kit for Blood (200 μl) and 1-step DNA Isolation Kit for Bacteria. The reason for selecting Nexxtec kits was based on the potential advantages of reduced potential contamination, having a single step between the digestion of the sample and the final DNA elution. All extractions were carried out following the manufacturers’ instructions, with the only modification of reducing the elution volume to 50 μL. 36 samples in total were used for extraction kit comparison, 12 per each of the three water bodies, 3 per each of the four different extraction kits tests, with two replicates for PCR and qPCR amplification (Table D in S1 File).

### PCR amplification

In order to overcome the potential specificity bias of species-specific primers [47] and to avoid differences based on single species representation in different lentic and lotic bodies, we used the vertebrate specific primer pair 12S-V5 developed by Riaz et al. [48], which amplifies a 144-bp long fragment of the 12s rRNA mtDNA gene and has been widely used [49–51]. The amplification reaction was performed in a total volume of 30 μl with, 12.5 μL Bioline BioMix Red PCR Mastermix (2X), 3 μL template, 1.5 μL of each primer (10 μM), adding sterile nuclease- free water to the final total volume. PCR conditions were as follow, 10 min at 95°C, followed by 40 cycles of 10 s at 95°C and 30 s at 52°C 30 s and 72°C for 30 s, with a final extension step at the 72°C for 5 min. PCR products were visualised on a 2% agarose gel and quantified with Qubit 1.0 fluorometer. Positive controls were used for the evaluation of primer pair efficiency with DNA extracted from two different fish species commonly found in Tawe and Cardiff Bay, brown trout (*Salmo trutta*) and Atlantic salmon (*Salmo salar*). DNA was extracted from muscle or fin tissue from these target species using the Qiagen DNeasy Blood & Tissue DNA extraction kit. A negative control PCR with no DNA template was added at all PCR amplifications.

SYBR Green technology (Bio-Rad, US) was used in real-time PCR in a combination with 12S-V5 primer pair in a final reaction volume of 20 μl which included, 10 μL SsoAdvanced Universal SYBR Green Supermix (1x), 3 μL template, 0.4 μL of each 12S-V5 primer (10 μM) and 6.2 μL sterile nuclease- free water. The qPCR amplification was performed under the following conditions: 7 min at 95°C, followed by 40 cycles of 10 s at 95°C and 30 s at 59°C. Each of one of the three sampling replicates was amplified twice on a plate and final average Cq values of the duplicates was used for the statistical analysis. Each qPCR plate included three negative controls consisting of sterile nuclease- free water instead of the template. A standard curve with 8- point 10- fold dilutions with starting concentration of 1 ng/ μL of genomic *Salmo trutta* DNA was used. *S*. *trutta* was chosen for the standard curve as it represents one of the most common fish species in Welsh freshwater bodies [52].

### Cloning

For species confirmation, four randomly selected samples from each water body and experimental design (twelve in total) were chosen and amplified with the 12S-V5 vertebrate primer pair using the same PCR protocol as above. The amplified PCR products (144 bp) were cloned into a pDRIVE Cloning Vector using Qiagen PCR cloning plus kit (Qiagen GmbH, Hilden, Germany) following the manufacturer's recommendations. Three different concentrations of ligation- reaction mixture were plated on agar plates: 20 μL, 50 μL and 100 μL. Plasmid DNA was extracted using the Wizard Plus SV Minipreps DNA Purification kit (Promega, Madison, WI, USA). Sequencing was then carried out with T7 and Sp6 primers at the Institute of Biological, Environmental and Rural Sciences (IBERS), Aberystwyth. For sequencing 12, 7 and 12 clones were randomly selected from the river Tawe, the pond and Cardiff Bay respectively, with lower representation of pond samples due to low number of colonies.

### Statistical analysis

For each response variable, eDNA capture yield (ng/ μL), PCR (ng/ μL) and qPCR (Ct values) amplification yields, three individual linear models were applied for each of the three experiments separately. For the volume experiment, volume and water body, including the interaction between them were used as predictors. For the filtration type experiment, water body and filtration type, were used as predictors including the interactions among them. For the extraction kit experiment, water body and extraction kit were used as predictors, including their interaction between them. Models with and without interactions between predictors for each of the three response variables (eDNA capture, PCR and qPCR) were compared based on AIC criteria using the ‘mass’ package. For the post-hoc analysis the ‘lsmeans’ package was used [53] based on Tukey contrasts for each experiment individually. Technical PCR and qPCR replicates were averaged before the analysis. Only samples with two technical working replicates were considered for further statistical analyses. Positive PCR and qPCR reactions without quantified DNA capture yield were only used for further comparison based on amplification efficiencies, excluding DNA yield. All statistical analyses were done with R, version 3.3.2.

## Results

In total 108 samples were extracted from all three freshwater bodies excluding negative and positive controls. No amplification appeared in filtration and extraction negative controls during PCR and qPCR. All positive controls performed as expected, and species were confirmed by Sanger sequencing of 144 bp length products. R^2^ values for the qPCR standard curve ranged from 0.95 to 1.00, and the efficiency ranged from 97 to 104%, with a slope between—3.3, -3.2. Average capture and amplification success rate including standard deviations for all three experiments presented separately for the DNA capture yield, PCR and qPCR efficiency are in Tables 1–3.

**Table 1:** Linear models analysing effects of filtration volume, filtration type and extraction kit in correlation to water body type on successful eDNA extraction and amplification for each of the experimental category separately, including comparison between models with and without an interaction term between the tested categories and water bodies.

Model	Dependent variable	Predictors	Model output statistics	AIC
**Volume experiment**				
DNA capture efficiency = Volume * Water body	DNA capture yield (ng/μ*L*)	Volume x Water body	F (8, 30) = 3.781, **p = 0.003**	-51.37
		Water body	F (2, 38) = 4.441, **p = 0.020**	
		Volume	F (4, 40) = 2.137, **p < 0.001**	
PCR efficiency = Volume * Water body	PCR efficiency (ng/μ*L*)	Volume x Water body	F (8, 37) = 1.327, p = 0.275	237.8
		Water body	F (2, 35) = 1.073, p = 0.356	
		Volume	F (4, 37) = 6.447, **p < 0.001**	
PCR efficiency = Volume	PCR efficiency (ng/μ*L*)	Volume	F (4, 37) = 6.049, **p < 0.001**	233.96
qPCR efficiency = Volume * Water body	qPCR (Cq values)	Volume x Water body	F (8, 24) = 1.167, p = 0.359	160
		Water body	F (2, 32) = 1.722, p = 0.2	
		Volume	F (4, 34) = 3.602, **p = 0.019**	
qPCR efficiency = Volume	qPCR (Cq values)	Volume	F (4, 34) = 3.330, **p = 0.020**	156.83
**Filtration type experiment**				
DNA capture efficiency = Filtration type * Water body	DNA capture yield (ng/μ*L*)	Filtration type X Water body	F (4,24) = 2.287, p = 0.105	-87.53
		Filtration type	F (2,24) = 4.294, **p = 0.032**	
		Water body	F (2,24) = 1.402, p = 0.274	
DNA capture efficiency = Filtration type	DNA capture yield (ng/μ*L*)	Filtration type	F (2,24) = 3.379, **p = 0.05**	-85.87
PCR efficiency = Filtration type* Water body	PCR efficiency (ng/μ*L*)	Filtration type X Water body	F (4,25) = 0.737, p = 0.580	140.23
		Water body	F (2,25) = 0.544, p = 0.590	
		Filtration type	F (2,25) = 3.990, **p = 0.037**	
PCR efficiency = Filtration type	PCR efficiency (ng/μ*L*)	Filtration type	F (2,25) = 4.362, **p = 0.024**	133.76
qPCR efficiency = Filtration type* Water body	qPCR (Cq values)	Filtration type X Water body	F (4,25) = 3.667, **p = 0.024**	101.37
		Water body	F (2,25) = 3.365, p = 0.058	
		Filtration type	F (2,25) = 5.845, **p = 0.011**	
qPCR efficiency = Filtration type	qPCR (Cq values)	Filtration type	F (2,25) = 3.501, **p = 0.047**	110.56
**Extraction kit experiment**				
DNA capture efficiency = Extraction kit * Water body	DNA capture yield (ng/μ*L*)	Extraction kit X Water body	F (6, 20) = 2.363, p = 0.069	-45.65
		Water body	F (2, 26) = 7.065, **p = 0.005**	
		Extraction kit	F (3, 29) = 10.657, **p = 0.001**	
PCR efficiency = Extraction kit * Water body	PCR efficiency (ng/μ*L*)	Extraction kit X Water body	F (6, 22) = 2.162, p = 0.086	187.1
		Water body	F (2, 28) = 6.412, **p = 0.006**	
		Extraction kit	F (3, 30) = 4.159, **p = 0.018**	
qPCR efficiency = Extraction kit * Water body	qPCR (Cq values)	Extraction kit X Water body	F (6, 20) = 2.042, p = 0.107	133.7
		Water body	F (2, 26) = 3.380, p = 0.054	
		Extraction kit	F (3, 28) = 0.299, p = 0.825	

**Table 2:** Comparison of water volumes by eDNA capture and amplification efficiencies for volume experiment using glass fibre filter (0.6 μm) and Qiagen extraction kit for each individual response, DNA capture yield (ng/ μL), PCR (ng/ μL) and qPCR (Cq).

Water body (n. of samples)	Volume	Mean DNA capture yield (±SD) (ng/μL)	Mean PCR efficiency (±SD) (ng/μL)	Mean qPCR efficiency (±SD) (Cq)
Cardiff Bay (15), Pond (15), Tawe (15)	15 mL*	0.027 ± 0.009	12.738 ± 4.203	32.978 ± 1.896
	100 mL	0.044 ± 0.045	8.813 ± 3.383	34.194 ± 1.236
	250 mL	0.040 ± 0.019	8.156 ± 4.797	33.960 ± 1.983
	1000 mL	0.087 ± 0.131	13.386 ± 1.793	33.683 ± 1.893
	2000 mL	0.406 ± 0.497	15.111 ± 2.473	31.242 ± 0.699

*The 15 mL volume within the volume experiment is based solely on ethanol- sodium acetate precipitation. Water bodies (Cardiff Bay, Tawe river and Pond) including number of sampling replicates per water body (15), total number of samples (45) and categories tested (15, 100, 250, 1000 and 2000 mL) are stated.

**Table 3:** Comparison of filtration methods for eDNA capture and amplification efficiencies for filtration type experiment for 100 mL water filtered using Qiagen extraction kit for each individual response DNA capture yield (ng/ μL), PCR (ng/ μL) and qPCR (Cq).

Water body (n. of samples)	Filtration type	Mean DNA capture yield (±SD) (ng/μL)	Mean PCR efficiency (±SD) (ng/μL)	Mean qPCR efficiency (±SD) (Cq)
Cardiff Bay (9), Pond (9), Tawe (9)	Cellulose nitrate	0.023 ±0.019	8.645 ± 1.207	35.626 ±2.341
	Glass fibre filter	0.022 ± 0.013	9.280 ± 3.293	34.115 ±1.157
	Syringe filtration + precipitation	0.070 ±0.058	12.593 ± 3.455	33.253 ±1.925

Water bodies (Cardiff Bay, Tawe river and Pond) including number of sampling replicates per water body (9), total number of samples (27) and categories tested (cellulose nitrate, glass fibre filter and syringe filtration + ethanol–sodium acetate precipitation) are stated.

Linear models found differences among water bodies, based on DNA capture efficiency (ng/ μL), for two out of the three individual experiments, water volume (F (2, 38) = 4.441, p = 0.020) and extraction kit (F (2, 26) = 7.065, p = 0.005), but not for the filtration type (F (2, 24) = 1.402, p = 0.274) (Table 1). Amplification rates in general did not differ between the water bodies for all of the linear models, with the following exception: PCR amplification in the extraction kit experiment (Table 1, F (2, 28) = 6.412, p = 0.006). Significant interactions were identified between water body and volume (F (8, 40) = 3.781, p = 0.003), with 2000 mL of water resulting in higher capture efficiency in the pond compared to all lower filtering volumes in the Tawe (Tukey's Post-hoc test, p < 0.001), and (based on qPCR) between filter type and water body (F (4, 25) = 3.667, p = 0.024), with much higher efficiency of syringe filtration combined with ethanol precipitation compared to cellulose nitrate filtering in the pond (Tukey's Post-hoc test, p < 0.001) (Table 1). Full linear models are reported in Table 1.

### Water filtration volume comparison

**Fig 2:**
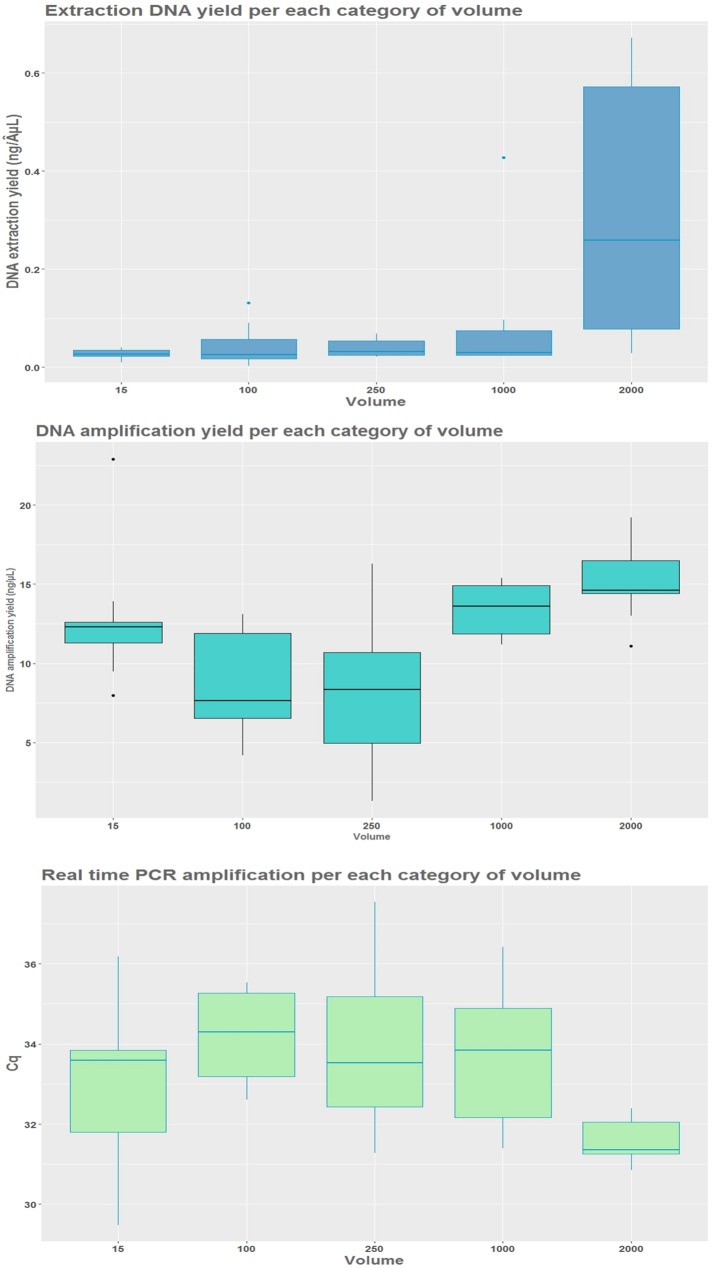
eDNA capture yield (ng/ μL) and amplification efficiencies by filtration volume experiment.

In total 45 samples (15 per water body corresponding to three sampling replicates for each one of the five-volume categories) were processed (Table 2), of which all samples were used for the statistical analyses for DNA capture yield, 42 samples for the PCR and 39 samples for the qPCR. eDNA capture yield increased with increase in filtered volume (F (4, 40) = 2.137, p < 0.001), with the highest DNA yield obtained from 2000 mL water sampled (Tukey's Post-hoc test, p < 0.001). There were significant differences between volumes for both amplifications (PCR, F (4, 37) = 6.049, p < 0.001; qPCR, F (4, 34) = 3.330, p = 0.020) with the most efficient qPCR amplification, being at 2000 mL of water filtered compared to 100 mL and 250 mL (Tukey's Post-hoc test, p = 0.010). The largest water volume filtered for the duration of experiment (2 L) showed the highest DNA capture efficiency (0.406 ± 0.497 ng/ μL), about tenfold higher compared to the other methods, followed by the 1 L (Fig 2). The capture yield for the 15 mL category was low compared to filtration with only 0.027 ± 0.009 ng/ μL yield (Table 2). There was a gradual increase in the eDNA capture efficiency from smallest 100 mL category up to largest, 2 L. The PCR amplification rate was the highest for the largest filtered volume tested with an average of 15.111 ± 2.473 ng/ μL (Table 2). The amplification rate for the 15 mL ethanol-sodium acetate precipitation method was high compared to other filtered volumes (12.738 ± 4.203 ng/ μL). The qPCR amplification efficiency for the 2 L category resulted in an average of 31.242 ± 0.699 cycles, comparatively similar to the 15 mL category with an average of 32.978 ± 1.896 cycles (Table 2).

Differences in eDNA capture yield (ng/ **μ**L), and amplification efficiencies by PCR (ng/ **μ**L) and qPCR (Cq) in the five different categorical groups for filtration volume experiment (15 mL, 100 mL, 250 mL, 1000 mL and 2000 mL), where each category is represented by three sampling replicates per three water bodies (9). For the amplification efficiency the technical duplicates of each sampling replicate were averaged before plotting. The 15 mL volume is based on ethanol- sodium acetate precipitation whereas the rest are based on water filtration. Filtration took place using glass fibre filter and eDNA was extracted with the Qiagen extraction kit.

### Filtration type comparison

**Fig 3:**
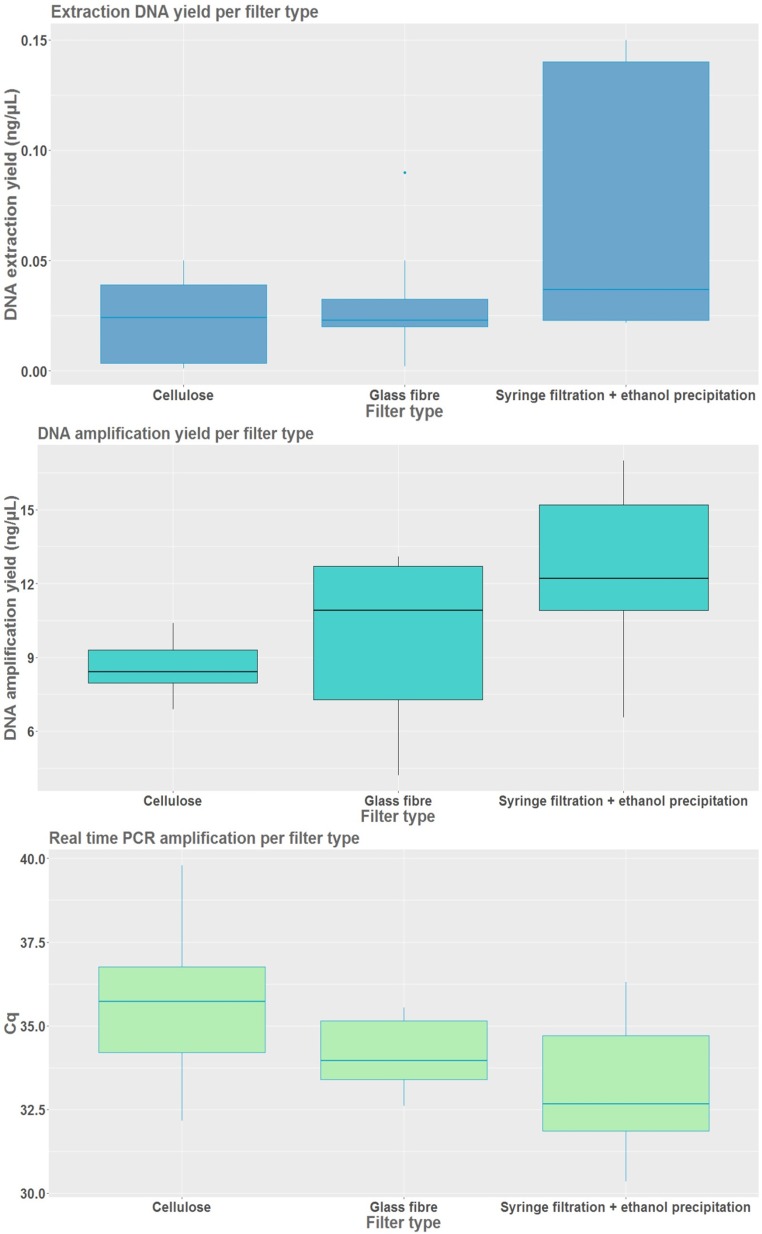
Filtration type experiment evaluating eDNA capture yield (ng/μL) and amplification efficiencies.

Twenty-seven samples were used for the comparison between the filtration types excluding three negative filtrations and one extraction controls, with nine samples representing each individual water body, as sample triplicates for each of the three individual filter types were examined (Table 3). For statistical analysis 25 samples were evaluated for DNA capture yield, 26 for PCR and 26 for qPCR. We found statistically significant differences between filtration type categories for DNA capture yield (F (2, 24) = 4.294, p = 0.032), PCR amplification (F (2, 25) = 4.362, p = 0.024) and qPCR (F (2, 25) = 5.845, p = 0.011). DNA extraction yield was the highest for the ethanol- sodium acetate precipitation in combination with filtration (0.070 ± 0.058 ng/ μL) in comparison to other two solely filtration procedures (Table 3). Both, filtration only methods without ethanol–sodium acetate precipitation performed poorly measured by DNA capture yield (Fig 3). Cellulose nitrate filters were the only filtration method where some of the filters failed to yield any eDNA and those samples were excluded from further statistical analysis (Table C in S1 File). PCR amplification efficiency using the combined method of syringe filtration and precipitation yielded the highest DNA concentrations (average value of 12.593 ± 3.45 ng/ μL) (Table 3). A slightly better amplification performance was produced by glass fibre filter (average value of 9.280 ± 3.293 ng/ μL) in comparison to cellulose nitrate filter with an average of 0.635 ng/ μL lower amplification rate. The syringe filtration in a combination with ethanol- sodium acetate precipitation resulted in low Cq values with an average of 33.235 ± 1.925 cycles evaluated by qPCR. QPCR provided similar results to PCR regarding performance of the glass fibre filter versus the cellulose nitrate filter with an average of 1.511 cycles higher for the glass fibre filter (Table 3).

Differences in eDNA capture as well as amplification by PCR and qPCR using three different filtration methods (C- Cellulose nitrate filter, G- Glass fibre filter, S- Syringe filter with a combination of ethanol- sodium acetate precipitation). Each experimental category is represented by three sampling replicates per three water bodies (9). For the amplification efficiencies the technical duplicates of each sampling replicate were averaged before plotting. The lowest Cq value corresponds to the highest efficiency.

### Extraction kit comparison

**Fig 4:**
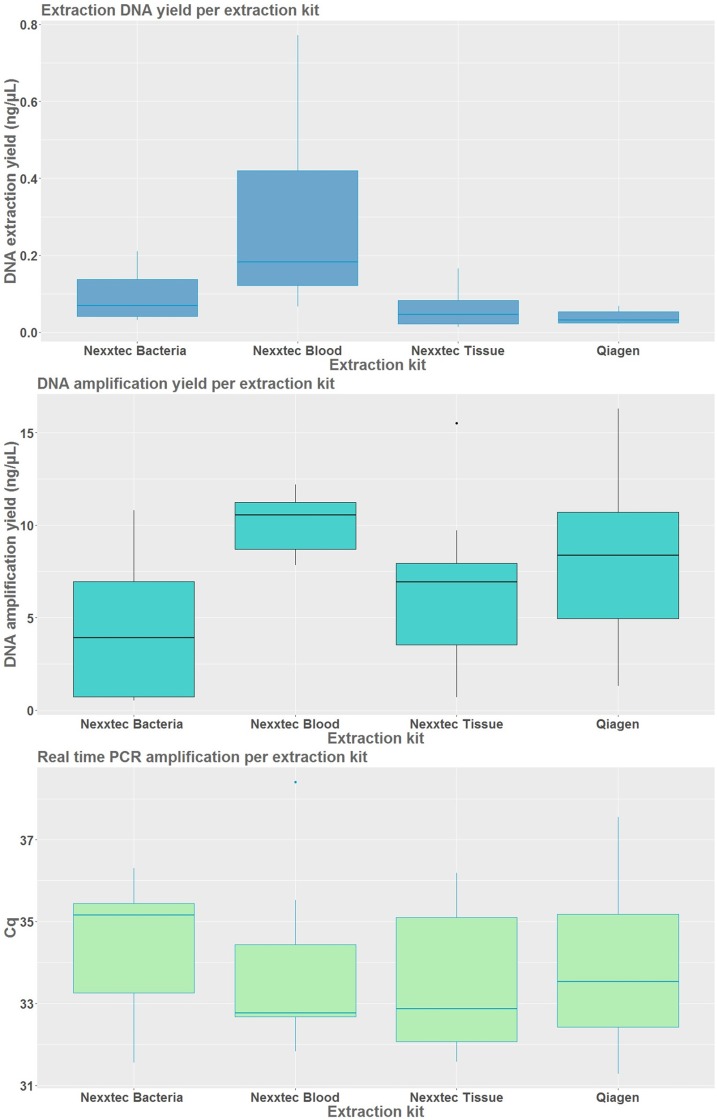
eDNA capture yield (ng/ μL) and amplification efficiencies by extraction kit comparison.

Thirty-six samples were extracted for the comparison between the filtration types, as well as one negative filtration and four negative extraction controls for each of the extraction kits tested (Table 4). Of these, twelve samples were used for each individual water body as sample triplicates were used for each individual extraction kit. In total, the statistical analysis based on DNA capture yield used 32 samples, 34 for the PCR and 32 for the qPCR. A model including both, the experimental groups and water bodies, identified significant differences between extraction kits for DNA capture yield (F (3, 29) = 10.657, p = 0.001) and PCR amplification (F (3, 30) = 4.159, p = 0.018), with the highest capture and amplification rate corresponding to the Nexxtec Blood kit (Tukey's Post-hoc test, p < 0.001), but there were no significant differences for qPCR (F (3, 28) = 0.299, p = 0.825) (Table 1). All Nexxtec kits were generally more efficient with regards to DNA capture in comparison to Qiagen (Fig 4). Between the Nexxtec kits the most efficient one appears to be the kit designed for blood samples with much higher efficiency compared to other two, 0.206 ng/ μL higher DNA capture yield on average. The 1—step Nexxtec DNA Isolation Kit for Blood proved particularly efficient with samples from Cardiff Bay with DNA capture yields of 0.511 ± 0.229 ng/ μL and had on average 4.438 ng/ μL higher amplification rate compared to other Nexxtec kits (Table 4).

**Table 4:** Comparison of extraction kits for eDNA capture and amplification efficiencies for extraction kit experiment with 250 ml of water filtered using a glass fibre filter (0.6 μm) for each individual response DNA capture yield (ng/ μL), PCR (ng/ μL) and qPCR (Cq).

Water body (n. of samples)	Extraction kit	Mean DNA capture yield (±SD) (ng/μL)	Mean PCR efficiency (±SD) (ng/μL)	Mean qPCR efficiency (±SD) (Cq)
Cardiff Bay (12), Pond (12), Tawe (12)	Nexxtec Blood	0.284 ±0.232	10.080 ±1.603	33.929 ±2.045
	Nexxtec Bacteria	0.095 ±0.068	4.784 ±4.133	34.392 ±1.841
	Nexxtec Tissue	0.061 ±0.051	6.607 ±4.721	33.551 ±1.848
	Qiagen	0.039 ±0.018	8.156 ±4.797	33.949 ±1.975

Water body (Cardiff Bay, Tawe River and Pond), number of sampling replicates per water body (12), total number of samples = 36 and extraction kits tested (Nexxtec Blood, Nexxtec Bacteria, Nexxtec Tissue and Qiagen).

Estimation of DNA capture extraction efficiency and amplification evaluated by PCR and qPCR, comparing between the following extraction kits Nexxtec bacteria, Nexxtec blood, Nexxtec Tissue and Qiagen. Each experimental category is represented by three sampling replicates per three water bodies (12). For the amplification efficiencies the technical duplicates of each sampling replicate were averaged before plotting.

### Species composition

**Fig 5:**
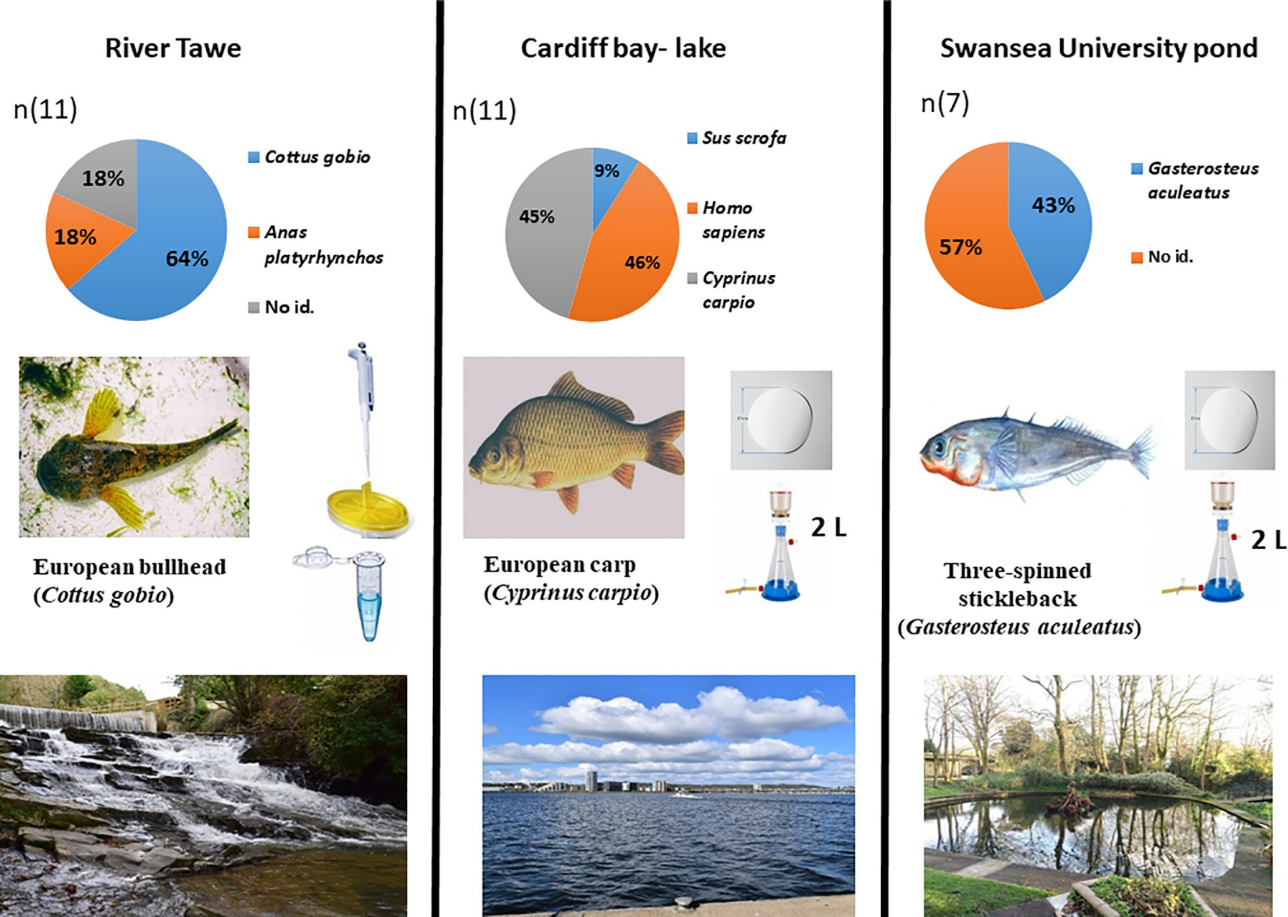
Graphical representation of the most successful sampling method for each specific water body: River Tawe, Cardiff Bay lake and Swansea University pond indicating the most common fish species identified.

Sequencing of the cloned PCR products indicated that the three dominant fish species found in each individual water body were European bullhead (*Cottus gobio*) in the Tawe River, three-spinned stickleback (*Gasterosteus aculeatus*) in the pond and European carp (*Cyprinus carpio*) in Cardiff Bay, irrespective of the sampling technique used (Fig 5, Table A in S1 File). As 12S-V5 are vertebrate primers, there were also human (*Homo sapiens)*, domestic pig (*Sus scrofa domesticus)* and common mallard (*Anas platyrhynchos*) sequences among the results. From 11 clones in the River Tawe, seven belonged to European bullhead, two were identified as *Anas platyrhynchos* and two remained unidentified. In the pond, three sequences belonged to *Gasterosteus aculeatus* and four remaining cloning sequences remained unidentified. In Cardiff Bay, five sequences belonged to *Cyprinus carpio*, five to *Homo sapiens*, and one to *Sus scrofa domesticus*.

Graphical representation of the most successful sampling method for all response variables tested for each water body separately. Pie charts indicate species proportion from total number (n) of sequenced cloned samples, River Tawe (11), lake Cardiff Bay (11) and Swansea University pond (7). The sequences that were not identified are marked as (unknown). Only one fish species was identified by Sanger sequencing in each water body using general vertebrate primers. The 2000 mL filtered water volume and syringe filtering with ethanol- sodium acetate precipitation technique appeared to be the most successful accounting for both, DNA capture and amplification. The selected images of fish were collected from the websites (Wikimedia, Titu.do, Flickr) marked with permission for reuse and modifications. The images of water bodies are personal copyright material.

## Discussion

The results from three different comparisons testing the effects of filtration volume, filtration type and extraction procedure, evaluated by DNA capture yield and amplification efficiencies highlight the importance of selecting the appropriate sampling method due to their variable efficiencies. Our results showed differences in capture yield among the three freshwater bodies depending on the volume and extraction kit used. This study indicates that a consistent eDNA sampling procedure is needed to be used in different freshwater bodies for species detection and quantitative assessment to be comparable across them.

It has been previously shown that a combination of different capture and extraction methods can result in different success rates of eDNA metabarcoding for different target groups [20], using universal primers [3, 5, 54]. Our approach, a novel combination of ethanol- sodium acetate precipitation with filtration, worked well in lentic and lotic water bodies with a high efficiency, easiness of handling, low cost, low chances of contamination and practicality. This method can be a reliable tool for eDNA species-specific assessments, confirming presence/ absence of certain species [55], as well as a sampling approach to determine community composition based on metabarcoding.

We examined the influence of filtration volume, filtration type, filtration method and type of extraction kit on capture yield and amplification efficiencies. DNA capture as well as amplification appeared to be very sensitive to changes in the volume of water filtered, as previously reported [20]. The efficiency of eDNA capture yield and amplification success rate largely differed between volumes. It would thus be recommended to filter as much of the water as possible, although the size distribution of particles in the aquatic environment can be a crucial factor determining the filter pore size and consequently the most feasible volume of water to be filtered [56]. Size of filtered particles [56], contamination risk [55] and feasibility of the proposed sampling (depending on location and proximity to the laboratory), can result in practical limitations in the maximum amount of water that is possible to filter [56]. DNA capture efficiency is an important evaluator of sampling technique used as it reflects the presence of the whole DNA within the sample. Another important factor is the number of replicates used for each individual evaluation, as the differences between the sampling triplicates were the most obvious in the DNA capture yield, where the whole extracted DNA, not just vertebrate eDNA, was quantified. In relation to this, while the highest eDNA capture rate corresponded to Cardiff Bay during the extraction kit experiment, it had the lowest amplification success (PCR and qPCR), potentially explained by high abundance of non- vertebrate DNA. This variability could be due to lack of power and more replicates would be recommended to increase reproducibility.

The most commonly used filter materials in eDNA studies are glass fibre filters [26, 31, 35] and cellulose nitrate filters [29, 32, 37] with different pore sizes, where larger pore sizes allow larger filtered water volumes and smaller pore sizes capture more particles but limit volume and speed of filtration [56]. Glass fibre filter with larger pore size resulted in slightly higher amplification efficiency compared to cellulose nitrate filter with smaller pore size, a contrasting result to previous ones, where normally larger filter pore size required larger volumes of water filtered [22]. The choice for the material of the filter type used depends as well on the practicality of usage during DNA extraction as filtration materials differ greatly. Judging by the easiness of filter handling and sample preparation for extraction, glass fibre material was preferred here. The difference between material types was not as high as the difference between capture techniques, with much higher efficiency of ethanol- sodium acetate precipitation in combination with filtration compared to filtration only.

The smallest water volume tested, based only on ethanol- sodium acetate precipitation (15 mL) provided solid amplification rates despite small volume. Thus, the newly proposed 100 mL syringe filtration with ethanol- sodium acetate precipitation method combines the strength of both techniques: the portability and easiness of the ethanol- sodium acetate precipitation while increasing the volume filtered and decreasing contamination risk by minimising filter handling. The proposed syringe filtration method appears to be highly efficient, affordable and reliable and it is thus an upgraded method from the one proposed by [30]. High efficiency of syringe filters compared to other filtration techniques has been shown with the use of Sterivex-GP polyethersulfone syringe filters, but it is a more costly alternative to the syringe filters used in this study [22].

The extraction kit seems to be the least important factor when it comes to selection of sampling techniques for eDNA capture. On several occasions extraction procedures based on usage of commercial kits resulted in no differences [25], whereas in other cases there has been shown significant variations [20, 23, 25]. The higher DNA capture efficiency and PCR amplification rates were provided by Nexxtec Blood kit, for which a single step before DNA elution highly minimises risk for the contamination. There was no difference between the extraction kits based on capture rate and the qPCR assessment.

Species- specific sequencing identified one dominant fish species per water body independently of the sampling technique used. This is likely due to the number of samples cloned only detecting the most abundant species in each area (Wharf Angling Club, 2018). Cardiff Bay is highly associated to human activities and the presence of human DNA is therefore not surprising. Mammal (including human) and avian DNA presence is common in eDNA studies utilising universal primers [57, 58] and all our negative filtration, extraction and PCR controls ensured that its origin was not laboratory contamination.

Our study contributes towards the understanding of the role of different sampling and extraction methods on the efficiencies of eDNA capture techniques. Focusing on well-known vertebrate primers [49–51, 59, 60] to avoid species-specific bias allowed us to compare efficiencies in three different water bodies with distinctive community composition, that can potentially introduce drawbacks assessing eDNA presence/ absence using qPCR, with a preferred species specific assay design. There was no difference between the PCR or qPCR success rate for the two most evident differential factors, the water bodies and volume, whereas filtration type and extraction kit differed greatly. Dissimilarities between capture and extraction techniques between pond, lake and river, highlight the importance of other abiotic aspects affecting eDNA yield such as acidity, substrate material and hydrological dynamics [39, 61, 62], including seasonality [63], which can majorly affect eDNA detection rates in different situations.

In summary, our study indicates that the main source of variation in the eDNA capture and amplification efficiencies is the sampling technique. Our results indicate that a careful sampling plan selecting the most efficient eDNA sampling protocol is essential and suggest that sampling and filtering the largest feasible volume is the best strategy. However, a syringe filtration through a 0.45 μm cellulose filter, combined with ethanol- sodium acetate precipitation is an alternative with low contamination risk/ high yield that can be easily used both in lotic and lentic environments with minimal sampling effort.

## Supporting information

S1 File qPCR melt curve plots of all three experiments volume, filter type and extraction kit carrier out in Tawe river (Figure A). qPCR melt curve plots of all three experiments volume, filter type and extraction kit carrier out in Cardiff Bay (Figure B). qPCR melt curve plots of all three experiments volume, filter type and extraction kit carrier out in Swansea University pond (Figure C). Identification of species in each of the water bodies pond, lake and river defined by capture and extraction technique, based on cloning and Sanger sequencing (Table A). Dataset for the filtration volume experiment combining all sampling triplicates from all three water bodies used for the statistical analysis, based on glass fibre filtration and Qiagen extraction kit (Table B). Dataset for the filtration type experiment combining all sampling triplicates from all three water bodies used for the statistical analysis, based on 100 mL filtered volume and Qiagen extraction kit (Table C). Dataset for the extraction kit experiment combining all sampling triplicates from all three water bodies used for the statistical analysis, based on 250 mL filtered volume using glass fibre filter (Table D).
(DOCX)
