# Supplementary material for: An optimised eDNA protocol for detecting fish in lentic and lotic freshwaters using a small water volume
Source: PLoS One. 2019 Jul 17;14(7):e0219218. doi: 10.1371/journal.pone.0219218 (PMC6636732; doi:10.1371/journal.pone.0219218)
Supplement: S1 File — qPCR melt curve plots of all three experiments volume, filter type and extraction kit carrier out in Tawe river (Figure A). qPCR melt curve plots of all three experiments volume, filter type and extraction kit carrier out in Cardiff Bay (Figure B). qPCR melt curve plots of all three experiments volume, filter type and extraction kit carrier out in Swansea University pond (Figure C). Identification of species in each of the water bodies pond, lake and river defined by capture and extraction technique, based on cloning and Sanger sequencing (Table A). Dataset for the filtration volume experiment combining all sampling triplicates from all three water bodies used for the statistical analysis, based on glass fibre filtration and Qiagen extraction kit (Table B). Dataset for the filtration type experiment combining all sampling triplicates from all three water bodies used for the statistical analysis, based on 100 mL filtered volume and Qiagen extraction kit (Table C). Dataset for the extraction kit experiment combining all sampling triplicates from all three water bodies used for the statistical analysis, based on 250 mL filtered volume using glass fibre filter (Table D). (DOCX) [file pone.0219218.s001.docx]

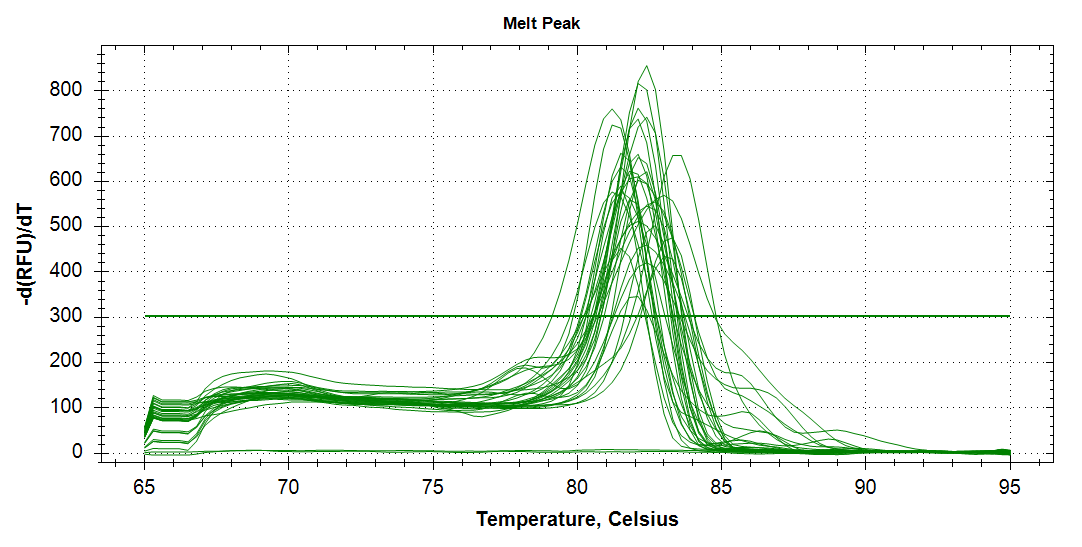


**Figure A**


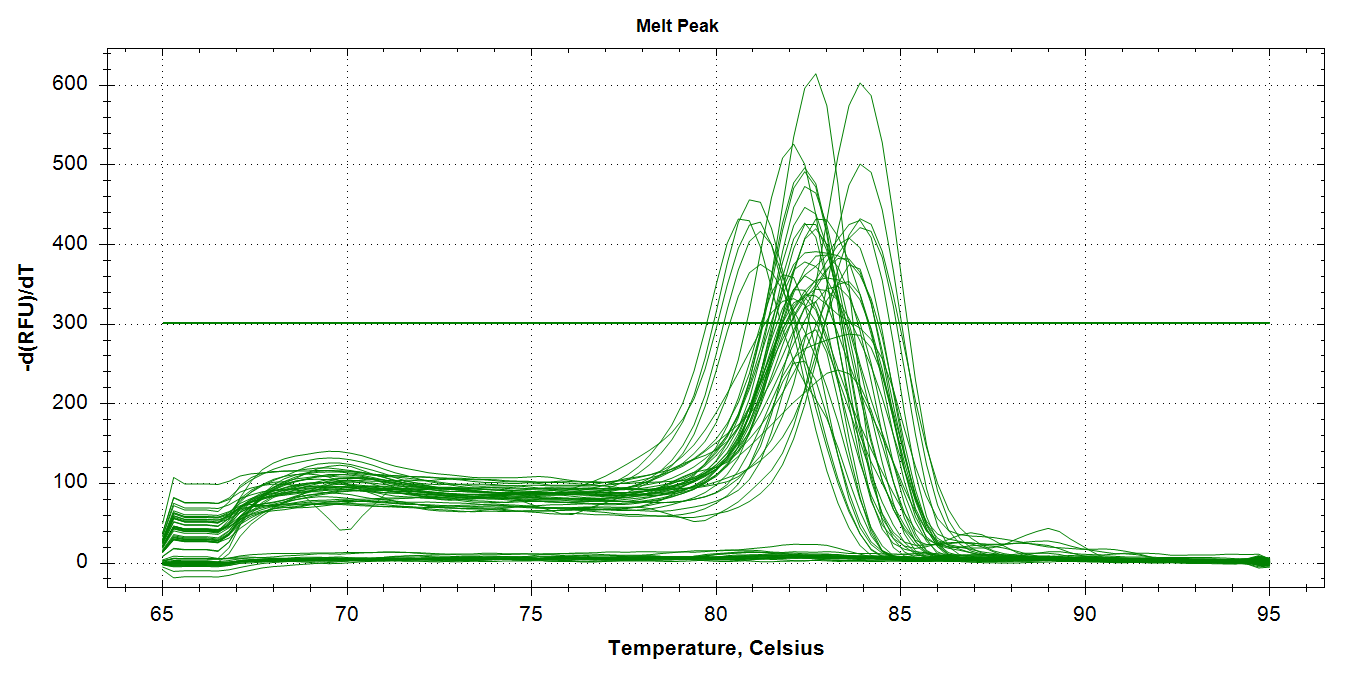


**Figure B**


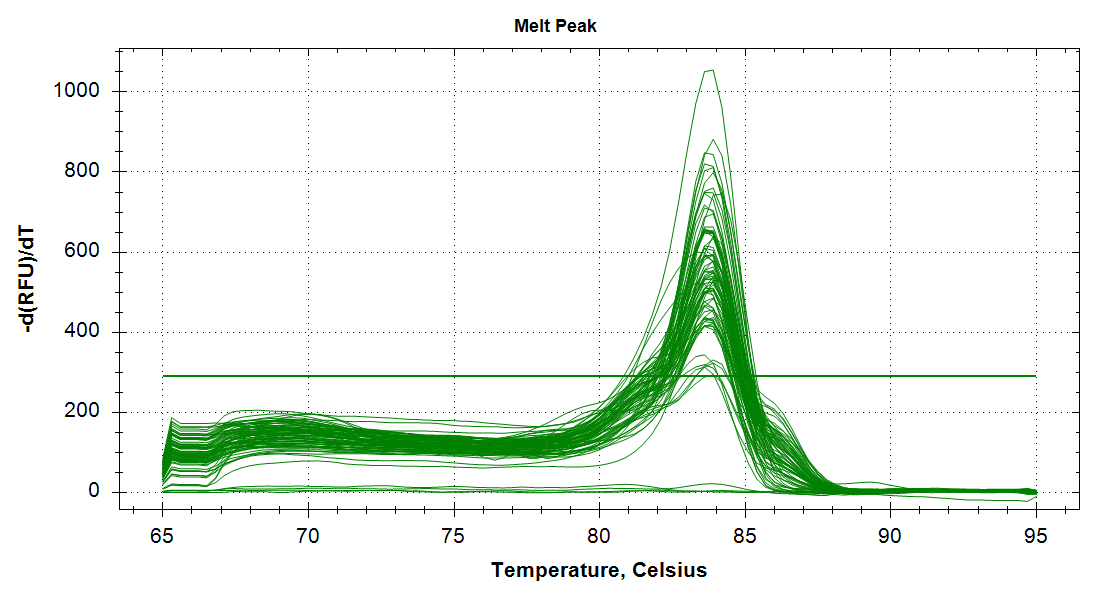


**Figure C**

**Table A**

| **Species** | **Experiment** | **Volume (mL)** | **Filter type** | **Extraction kit** | **Technique** | **Water body** |
| --- | --- | --- | --- | --- | --- | --- |
| *Cyprinus carpio* | Volume | 2000 | Glass fibre | Qiagen | Filtration | Lake |
| *Cyprinus carpio* | Filter type | 100 | Cellulose | Qiagen | Filtration | Lake |
| *Cyprinus carpio* | Extraction kit | 250 | Glass fibre | Nexxtec Blood | Filtration | Lake |
| *Cyprinus carpio* | Extraction kit | 250 | Glass fibre | Nexxtec Blood | Filtration | Lake |
| *Cyprinus carpio* | Volume | 100 | Glass fibre | Qiagen | Filtration | Lake |
| *Homo sapiens* | Volume | 100 | Glass fibre | Qiagen | Filtration | Lake |
| *Homo sapiens* | Volume | 2000 | Glass fibre | Qiagen | Filtration | Lake |
| *Homo sapiens* | Filter type | 100 | Cellulose | Qiagen | Filtration | Lake |
| *Homo sapiens* | Extraction kit | 250 | Glass fibre | Qiagen | Filtration | Lake |
| *Homo sapiens* | Extraction kit | 250 | Glass fibre | Qiagen | Filtration | Lake |
| *Sus scrofa domesticus* | Volume | 100 | Glass fibre | Qiagen | Filtration | Lake |
| *Anas platyrhynchos* | Extraction kit | 250 | Glass fibre | Nexxtec Tissue | Filtration | River |
| *Anas platyrhynchos* | Volume | 250 | Glass fibre | Qiagen | Filtration | River |
| No identification | Volume | 250 | Glass fibre | Qiagen | Filtration | River |
| No identification | Extraction kit | 250 | Glass fibre | Nexxtec Tissue | Filtration | River |
| *Cottus gobio* | Extraction kit | 250 | Glass fibre | Nexxtec Tissue | Filtration | River |
| *Cottus gobio* | Filter type | 100 | Cellulose | Qiagen | Ethanol precipitation | River |
| *Cottus gobio* | Filter type | 100 | Cellulose | Qiagen | Ethanol precipitation | River |
| *Cottus gobio* | Volume | 250 | Glass fibre | Qiagen | Filtration | River |
| *Cottus gobio* | Volume | 1000 | Glass fibre | Qiagen | Filtration | River |
| *Cottus gobio* | Volume | 1000 | Glass fibre | Qiagen | Filtration | River |
| *Cottus gobio* | Filter type | 100 | Cellulose | Qiagen | Ethanol precipitation | River |
| *Gasterosteus aculeatus* | Volume | 15 |  | Qiagen | Ethanol precipitation | Pond |
| *Gasterosteus aculeatus* | Extraction kit | 250 | Glass fibre | Nexxtec Tissue | Filtration | Pond |
| *Gasterosteus aculeatus* | Volume | 1000 | Glass fibre | Qiagen | Filtration | Pond |
| No identification | Volume | 1000 | Glass fibre | Qiagen | Filtration | Pond |
| No identification | Volume | 15 |  | Qiagen | Ethanol precipitation | Pond |
| No identification | Extraction kit | 250 | Glass fibre | Nexxtec Tissue | Filtration | Pond |
| No identification | Filter type | 100 | Cellulose | Qiagen | Ethanol precipitation | Pond |

**Table B**

| **Test Name** | **Volume** | | **Water body** | **DNA capture yield (ng/ µL)** | **PCR (ng/ µL)** | **qPCR (Cq)** |
| --- | --- | --- | --- | --- | --- | --- |
| A2 15 | 15 | Lake | | 0.0396 | 12.4 | 32.62 |
| A1 15 | 15 | Lake | | 0.0268 | 11.8 | 31.8 |
| A3 15 | 15 | Lake | | 0.0344 | 13.9 | 31.57 |
| A3 G | 100 | Lake | | 0.131 | 5.08 | NA |
| A1 G | 100 | Lake | | 0 | 12.7 | 33.6 |
| A2 G | 100 | Lake | | 0.09 | 7.64 | 33.59 |
| A3 250 | 250 | Lake | | 0.056 | 1.3 | 32.93 |
| A2 250 | 250 | Lake | | 0.068 | 4.96 | 32.33 |
| A1 250 | 250 | Lake | | 0.026 | 7 | 31.28 |
| A1 1L | 1000 | Lake | | 0.03 | 11.3 | NA |
| A3 1L | 1000 | Lake | | 0.0236 | 12.4 | 35.64 |
| A2 1L | 1000 | Lake | | 0.0232 | 14.4 | 31.92 |
| A2 2L | 2000 | Lake | | 0.062 | 14.6 | NA |
| A1 2L | 2000 | Lake | | 0.0288 | 14.4 | NA |
| A3 2L | 2000 | Lake | | 0.08 | 14.8 | 32.39 |
| P3 15 | 15 | Pond | | 0.03 | 7.96 | 33.85 |
| P1 15 | 15 | Pond | | 0.0224 | 12.6 | 33.84 |
| P2 15 | 15 | Pond | | 0.02 | 9.48 | 29.47 |
| P2 100 | 100 | Pond | | 0.02 | 10.9 | 35.54 |
| P1 100 | 100 | Pond | | 0.024 | 6.52 | 35.05 |
| P3 100 | 100 | Pond | | 0.022 | 13.1 | 34.3 |
| P3 250 | 250 | Pond | | 0 | 10.7 | 35.73 |
| P2 250 | 250 | Pond | | 0.0216 | 8.36 | 35.18 |
| P1 250 | 250 | Pond | | 0.032 | 16.3 | 32.43 |
| P3 1L | 1000 | Pond | | 0.0504 | NA | 36.42 |
| P2 1L | 1000 | Pond | | 0.024 | NA | 34.15 |
| P1 1L | 1000 | Pond | | 0.428 | NA | 31.4 |
| P1 2L | 2000 | Pond | | 0.323 | 11.1 | 31.38 |
| P2 2L | 2000 | Pond | | 0.672 | 14.4 | 31.23 |
| P3 2L | 2000 | Pond | | 0.9 | 19.2 | 30.27 |
| T3 15 | 15 | River | | 0.036 | 12.3 | 36.18 |
| T2 15 | 15 | River | | 0.01 | 11.3 | 33.88 |
| T1 15 | 15 | River | | 0.024 | 22.9 | 33.59 |
| T1 100 | 100 | River | | 0.002 | 4.2 | 35.5 |
| T2 100 | 100 | River | | 0.02 | 11.9 | 32.78 |
| T3 100 | 100 | River | | 0.046 | 7.28 | 32.6 |
| T3 250 | 250 | River | | 0.0516 | 13.3 | 37.55 |
| T1 250 | 250 | River | | 0 | 8.44 | 34.68 |
| T2 250 | 250 | River | | 0.0232 | 3.04 | 33.53 |
| T2 1L | 1000 | River | | 0.0744 | 15.4 | NA |
| T3 1L | 1000 | River | | 0.0292 | 15.4 | 33.84 |
| T1 1L | 1000 | River | | 0.0964 | 11.2 | 32.41 |
| T1 2L | 2000 | River | | 0.0776 | 16.5 | NA |
| T2 2L | 2000 | River | | 0.259 | 18 | 31.33 |
| T3 2L | 2000 | River | | 0.572 | 13 | 30.85 |

**Table C**

| **Test Name** | **Water body** | **Filter type** | **Pore size** | | **DNA capture yield (ng/ µL)** | **PCR (ng/ µL)** | **qPCR (Cq)** |
| --- | --- | --- | --- | --- | --- | --- | --- |
| A2 C | Lake | Cellulose | | Small | 0.026 | 6.88 | 32.16 |
| A1 C | Lake | Cellulose | | Small | 0.038 | 8 | 33.6 |
| A3 C | Lake | Cellulose | | Small | NA | NA |  |
| A1 100 | Lake | Glass fibre | | Large | 0.0267 | 12.7 | 33.6 |
| A2 100 | Lake | Glass fibre | | Large | 0.09 | 7.64 | 33.59 |
| A3 100 | Lake | Glass fibre | | Large | NA | 12.7 | 35.3 |
| A3 S | Lake | Syringe | | Small | 0.0228 | 11 | 32.28 |
| A1 S | Lake | Syringe | | Small | 0.0228 | 15.2 | 32.66 |
| A2 S | Lake | Syringe | | Small | 0.14 | 17 | 31.86 |
| P1 C | Pond | Cellulose | | Small | 0.001 | 8.8 | 35.19 |
| P2 C | Pond | Cellulose | | Small | 0.002 | 8.04 | 39.8 |
| P3 C | Pond | Cellulose | | Small | 0.0416 | 10.2 | 36.86 |
| P2 100 | Pond | Glass fibre | | Large | 0.02 | 10.9 | 35.54 |
| P3 100 | Pond | Glass fibre | | Large | 0.022 | 13.1 | 34.3 |
| P1 100 | Pond | Glass fibre | | Large | 0.024 | 6.52 | 35.05 |
| P3 S | Pond | Syringe | | Small | 0.0612 | 6.56 | 34.7 |
| P1 S | Pond | Syringe | | Small | 0.144 | 16.1 | 31.74 |
| P2 S | Pond | Syringe | | Small | 0.15 | 10.9 | 30.34 |
| T1 C | River | Cellulose | | Small | 0.004 | 10.4 | 36.26 |
| T3 C | River | Cellulose | | Small | 0.0224 | 9 | 36.73 |
| T2 C | River | Cellulose | | Small | 0.05 | 7.84 | 34.41 |
| T1 100 | River | Glass fibre | | Large | 0.002 | 4.2 | 35.46 |
| T2 100 | River | Glass fibre | | Large | 0.02 | 11.9 | 32.78 |
| T3 100 | River | Glass fibre | | Large | 0.05 | 7.28 | 32.6 |
| T3 S | River | Syringe | | Small | 0.0216 | 14.9 | 36.3 |
| T2 S | River | Syringe | | Small | 0.0272 | 9.48 | 34.9 |
| T1 S | River | Syringe | | Small | 0.0368 | 12.2 | 34.5 |

*****Syringe filtration with ethanol precipitation.

**Table D**

| **Test Name** | **Water body** | **Extraction kit** | **DNA capture yield (ng/ µL)** | **PCR (ng/ µL)** | **qPCR (Cq)** |
| --- | --- | --- | --- | --- | --- |
| A1 NG | Lake | Nexxtec Bacteria | 0.211 | 0.724 | NA |
| A2 NG | Lake | Nexxtec Bacteria | 0.127 | 6.96 | 31.56 |
| A3 NG | Lake | Nexxtec Bacteria | 0.0908 | 0.58 | NA |
| P1 NG | Pond | Nexxtec Bacteria | 0.0312 | 3.92 | NA |
| P2 NG | Pond | Nexxtec Bacteria | 0.0484 | 0.516 | 35.48 |
| P3 NG | Pond | Nexxtec Bacteria | 0.0424 | 2.64 | 34.96 |
| T1 NG | River | Nexxtec Bacteria | 0.168 | 10.8 | 3.27E+01 |
| T2 NG | River | Nexxtec Bacteria | 0.038 | 6.12 | 35.35 |
| T3 NG | River | Nexxtec Bacteria | NA | 10.8 | 36.3 |
| A1 NB | Lake | Nexxtec Blood | 0.42 | 8.12 | 38.41 |
| A2 NB | Lake | Nexxtec Blood | 0.343 | 11.3 | 34.44 |
| A3 NB | Lake | Nexxtec Blood | 0.772 | 7.84 | 31.83 |
| P1 NB | Pond | Nexxtec Blood | 0.128 | 10.5 | 32.77 |
| P2 NB | Pond | Nexxtec Blood | 0.122 | 11.2 | 32.68 |
| P3 NB | Pond | Nexxtec Blood | 0.436 | 10.6 | 34.34 |
| T1 NB | River | Nexxtec Blood | 0.183 | 8.88 | 32.76 |
| T2 NB | River | Nexxtec Blood | 0.0868 | 12.2 | 32.61 |
| T3 NB | River | Nexxtec Blood | 0.0672 | NA | 35.52 |
| A1 NT | Lake | Nexxtec Tissue | NA | NA | NA |
| A2 NT | Lake | Nexxtec Tissue | 0.166 | 0.704 | 32.1 |
| A3 NT | Lake | Nexxtec Tissue | 0.0788 | 1.53 | 31.58 |
| P1 NT | Pond | Nexxtec Tissue | 0.0488 | 15.5 | 33.03 |
| P2 NT | Pond | Nexxtec Tissue | 0.0448 | 6.8 | 32.72 |
| P3 NT | Pond | Nexxtec Tissue | 0.0924 | 7.04 | 31.99 |
| T1 NT | River | Nexxtec Tissue | 0.0132 | 9.72 | 3.48E+01 |
| T2 NT | River | Nexxtec Tissue | 0.0218 | 7.36 | 36.19 |
| T3 NT | River | Nexxtec Tissue | 0.0208 | 4.2 | 36 |
| A1 250 | Lake | Qiagen | 0.026 | 7 | 31.28 |
| A2 250 | Lake | Qiagen | 0.068 | 4.96 | 32.33 |
| A3 250 | Lake | Qiagen | 0.056 | 1.3 | 32.93 |
| P1 250 | Pond | Qiagen | 0.032 | 16.3 | 32.43 |
| P2 250 | Pond | Qiagen | 0.0216 | 8.36 | 35.18 |
| P3 250 | Pond | Qiagen | NA | 10.7 | 35.73 |
| T1 250 | River | Qiagen | NA | 8.44 | 34.68 |
| T2 250 | River | Qiagen | 0.0232 | 3.04 | 33.53 |
| T3 250 | River | Qiagen | 0.0516 | 13.3 | 37.55 |
